# Long-term hepatitis B infection in a scalable hepatic co-culture system

**DOI:** 10.1038/s41467-017-00200-8

**Published:** 2017-07-25

**Authors:** Benjamin Y. Winer, Tiffany S. Huang, Eitan Pludwinski, Brigitte Heller, Felix Wojcik, Gabriel E. Lipkowitz, Amit Parekh, Cheul Cho, Anil Shrirao, Tom W. Muir, Eric Novik, Alexander Ploss

**Affiliations:** 1 Department of Molecular Biology, Princeton University, Princeton, NJ 08544 USA; 2 Hurel® Corporation, North Brunswick, NJ 08902 USA; 3 Department of Chemistry, Princeton University, Princeton, NJ 08544 USA

## Abstract

Hepatitis B virus causes chronic infections in 250 million people worldwide. Chronic hepatitis B virus carriers are at risk of developing fibrosis, cirrhosis, and hepatocellular carcinoma. A prophylactic vaccine exists and currently available antivirals can suppress but rarely cure chronic infections. The study of hepatitis B virus and development of curative antivirals are hampered by a scarcity of models that mimic infection in a physiologically relevant, cellular context. Here, we show that cell-culture and patient-derived hepatitis B virus can establish persistent infection for over 30 days in a self-assembling, primary hepatocyte co-culture system. Importantly, infection can be established without antiviral immune suppression, and susceptibility is not donor dependent. The platform is scalable to microwell formats, and we provide proof-of-concept for its use in testing entry inhibitors and antiviral compounds.

## Abstract (web summary)

The lack of models that mimic hepatitis B virus (HBV) infection in a physiologically relevant context has hampered drug development. Here, Winer et al. establish a self-assembling, primary hepatocyte co-culture system that can be infected with patient-derived HBV without further modifications.

## Introduction

Hepatitis B virus (HBV) belongs to the *Hepadnaviridae* family and has a very compact, partially double-stranded 3.2 kb DNA genome known as relaxed circular DNA (rcDNA). HBV entry is dependent on the bile acid transporter human sodium-taurocholate cotransporting polypeptide (hNTCP), which is exclusively expressed in hepatocytes^1, 2^. Interaction of HBV surface antigen (HBsAg) with NTCP initiates uptake, during which the virus is internalized via receptor-mediated endocytosis (reviewed in ref. ^3^). Following uncoating, HBV rcDNA is transported and released into the nucleus. The rcDNA contains several DNA lesions and hijacks the liver DNA repair system to form a stable HBV DNA molecule^4, 5^. This DNA molecule is referred to as covalently closed circular DNA (cccDNA). cccDNA is a chromatinized “mini-chromosome” and serves as the transcriptional template for all four viral gene products—envelope (L, M, and S), core and X antigens (Ags) and the viral polymerase—as well as the pgRNA (pre-genomic RNA). pgRNA can be reverse transcribed into rcDNA, which can be enclosed by a lipid bilayer containing the HBV envelope proteins and then released from the host cell, thereby completing the HBV life cycle^6^.

HBV cccDNA is the cause of persistent HBV infection and subsequent severe liver disease, including hepatocellular carcinoma (HCC). In order to prevent HCC, it is imperative to purge or at least effectively silence cccDNA. Unfortunately, despite decade-long efforts, fundamental aspects of how cccDNA is formed, maintained and transcriptionally regulated remain opaque. Antivirals to cure chronic HBV, such as those that target cccDNA, have not been successfully generated. Development of such curative therapies has been hampered by the scarcity of experimental systems that recapitulate the chronic phase of the infection.

HBV has a narrow tissue and host tropism limited to productive infections in human and chimpanzee hepatocytes, posing challenges for the study of HBV in experimental models^4^. Transfection of plasmids encoding larger-than-genome-size HBV sequences into human hepatoma cells has facilitated the study of some aspects of the HBV life cycle^7, 8^. However, as an artificial system and not a bona fide infection, critical steps of the viral life cycle are not faithfully recapitulated. Other work has shown that specific cell lines derived from human HCCs, such as HepaRG cells, are susceptible to HBV^9^. The panel of cell lines that can be infected with HBV was substantially expanded after the identification of human NTCP, also known as SLC10A1, as a functional receptor for HBV and hepatitis delta virus (HDV)^1, 2^. Indeed, ectopic expression of human NTCP is sufficient to increase permissiveness in a variety of immortalized liver cells^1, 2^. Although experiments in hepatoma cell lines can be reproducible and inexpensive, these immortalized cells do not adequately recapitulate the physiological environment of primary hepatocytes due to their abnormal proliferation and aberrant gene regulation. For in vitro experiments, primary hepatocyte cultures are thus more desirable^10^. Previous work has indeed shown that primary human hepatocytes (PHHs) of both adult and fetal origin can be infected with HBV^11–16^. However, long-term infections of PHHs with HBV or other hepatotropic pathogens, such as hepatitis C virus (HCV) or parasites that cause malaria in humans, have been notoriously difficult due to their rapid dedifferentiation and loss of characteristic hepatic functions following isolation and plating. As a result, analyses of HBV’s interactions with the host cell have been largely limited to the first few days following plating, reflecting only acute infection. PHH dedifferentiation can be delayed/prevented in collagen sandwich cultures, by aggregation in spheroids or in co-culture with non-parenchymal cells^17, 18^. For the latter approach, both self-assembling (SACC) and micro-patterned PHH co-cultures (MPCC) are effective formats to stabilize hepatic function, especially if oxidative stress is reduced during the onset of the culture^19–21^. MPCC of PHHs and murine 3T3 fibroblasts have been infected with HBV, HCV, and *Plasmodium falciparum* and *vivax* ^22–24^. However, in these studies, HBV infection was limited to a few donors and required suppression of antiviral signaling, posing problems for studying host responses to HBV for antiviral drug testing^22^.

Here, we aimed to carefully characterize HBV infection in SACC-PHHs in culture formats amenable to high-throughput analysis. We demonstrate that SACC-PHHs generated with pooled or single PHH donors but not PHH monocultures support persistent infection with cell-culture and patient derived HBV for more than 40 days. The platform can be miniaturized to 96-well plate formats in which uniform HBV infection can be achieved. Using this high-throughput platform, we show that a preS1-derived myristoylated peptide can efficiently prevent HBV uptake, which is consistent with previous findings in vitro^25, 26^. Likewise, administration of a clinically approved inhibitor of the HBV polymerase, entecavir, suppressed HBV viremia in a dose-dependent fashion. In contrast, pharmacological inhibition of the host enzyme tyrosyl-DNA-phosphodiesterase 2 (TDP2), which has been implicated in a critical step prior to cccDNA formation^27^, had no effect on HBV infection. Collectively, our data establish proof-of-concept for the utility of the SACC-PHH platform as a versatile, robust platform to study HBV persistence of genetically diverse viral isolates and for efficacy assessments of host-targeting and directly acting antivirals.

## Results

### Persistent HBV infection in SACC-PHHs

**Fig. 1:**
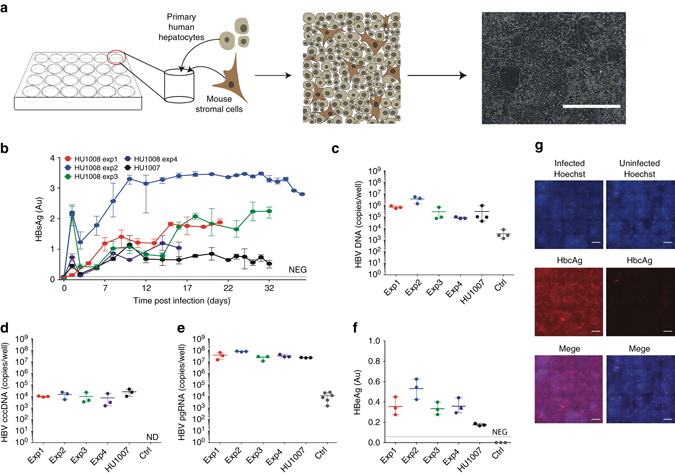
Persistent HBV infection of self-assembling primary hepatocyte co-cultures. **a** PHH platform. HBVcc infection of mixed PHH donors performed in five separate experiments with two separate lots of hepatocytes. HBsAg concentrations determined in the supernatants **b** and total HBV DNA **c**, HBV cccDNA **d**, HBV pgRNA **e** quantified in SACC-PHH lysates at the final HBsAg timepoint. **f** HBeAg was quantified in the supernatants (day 16 post infection) by HBeAg ELISA. **g** HBcAg detection in HBV-infected (*left panels*) and non-infected (*right panels*) mixed donor SACC-PHHs by immunofluorescence microscopy; HBcAg (*red*), nuclear Hoechst dye (*blue*), all scale bars are 200 μm. For all HBVcc infections of mixed PHH donors three to five biological replicates were performed. All data are presented as means ± s.d

SACCs are established by plating PHHs (Supplementary Table 1) with non-parenchymal stromal cells in collagen-coated tissue culture plates (Fig. 1a) utilizing a protocol previously reported to promote advanced hepatic morphology, such as bile caniliculi formation, and to enhance numerous hepato-specific functions for extended culture periods (Supplementary Table 2)^19, 28–30^.

To test susceptibility to HBV, SACC-PHHs were exposed to different amounts of purified, cell culture-derived HBV (HBVcc). HBV infection was considerably more robust in SACC-PHHs generated from pooled hepatocyte donors (donor characteristics summarized in Supplementary Tables 1 and 2) compared to a HepG2 cell clone (3B10) expressing high levels of hNTCP (Supplementary Fig. 1a–d) as evidenced by significantly greater secretion of HBsAg (Supplementary Fig. 2a, b). HBV infection was highly reproducible and not dependent on particular lots of pooled hepatocyte donors or batches of HBVcc inocula (Fig. 1b, c). Previous work found inhibition of Janus kinase (JAK)-dependent signaling necessary to establish HBVcc infection for up to 19 days in MPCCs^22^. In contrast, in SACC-PHHs, HBsAg secretion was sustained for more than 30 days post-infection without suppression of antiviral defenses (Fig. 1b). At the end point, HBV DNA (Fig. 1c), cccDNA (Fig. 1d, Supplementary Fig. 3 **)** and HBV pgRNA (Fig. 1e) were readily detected in lysates of HBV-infected SACC-PHHs. The detection of HBV precore antigen (HBeAg) in the HBVcc-challenged samples provided additional evidence that cccDNA was formed and that active viral transcription was occurring from the cccDNA template (Fig. 1f)^31^. Immunofluorescent visualization of HBV core antigen (HBcAg) demonstrated that the majority of hepatocytes in the culture were infected (Fig. 1g).

All infections were carried out between 7–10 days following plating at a point previously determined as optimal for restoring hepatic functions in the cultures^29, 32, 33^. To determine whether the time point of initial HBV infection could possibly be extended further, we challenged mixed donor SACC-PHHs with HBVcc 10, 15, or 20 days post-plating (schematic Supplementary Fig. 4a). We observed that the SACC-PHHs remained susceptible for all time points tested as indicated by HBsAg secretion. However, SACC-PHHs challenged at 10 and 15 days post seeding reached comparable levels of HBsAg 8 days after infection, cells that were challenged 20 days post seeding became infected but secreted substantially lower amounts of HBsAg indicative of a less efficient infection. This may indicate that either SACC-PHHs at day 20 post seeding became less susceptible to HBV infection or that transcriptional changes have occurred that affect HBsAg secretion (Supplementary Fig. 4b).

**Fig. 2:**
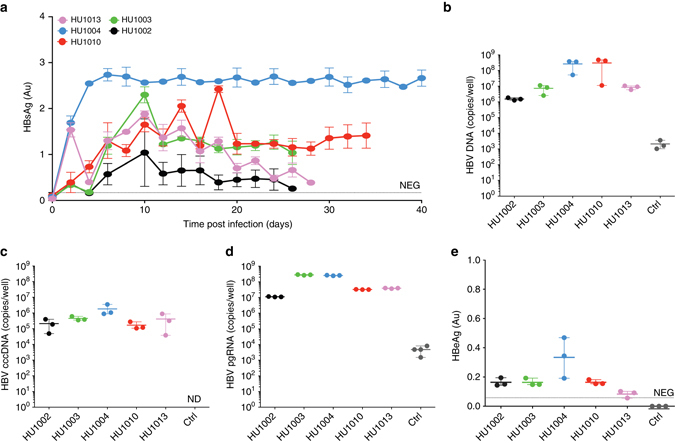
Persistent HBV infection in single donor PHH co-cultures. Assessment of HBVcc infection of single donor SACC-PHH co-cultures. Quantification of HBsAg concentrations in the supernatants **a**, and total HBV DNA **b**, cccDNA **c**, pgRNA **d** in SACC-PHH lysates. **e** HBeAg was quantified in the supernatants (day 16 post infection) by HBeAg ELISA. For all HBVcc infections of single PHH donors three to five biological replicates were performed. All data are presented as means ± s.d

To determine whether the robustness of the platform was in part due to the use of pooled donor PHH lots, we analyzed HBVcc infection in SACC-PHHs established from five single donors. HBsAg was detectable shortly after inoculation of the cultures with HBVcc and was sustained for the duration of the experiment (Fig. 2a). HBV DNA (Fig. 2b), cccDNA (Fig. 2c, Supplementary Fig. 3) and pgRNA (Fig. 2d) reached levels similar to those in HBVcc-infected SACC-PHHs generated with pooled donor lots. In line with these observations, HBeAg was detectable in all infected samples and largely corresponded with secreted HBsAg levels (Fig. 2e). To affirm that the high permissiveness of the PHHs in co-culture is attributable to the culture format, we directly compared susceptibility of SACC-PHHs with PHHs of the same hepatocyte donor (HU1003). The morphology of the monoculture after plating showed the classic cobblestone pattern of healthy PHHs (Supplementary Fig. 5a, *left*), but by day eight morphological changes had occurred indicating de-differentiation and deterioration of the culture (Supplementary Fig. 5a, *right*). This loss of hepatocyte morphology also correlated with a decrease in hepatic functions as evidenced by the progressively lower levels of secreted albumin (Supplementary Fig. 5b). As expected, exposure of PHH monocultures to HBVcc thus did not yield any measurable evidence of infection, in stark contrast to the high HBV permissiveness of the hepatocytes in the SACC-PHH format (Supplementary Fig. 5c).

### Persistent HBV infection using HBV patient isolates

HBV is a genetically diverse virus that has been classified into eight genotypes (A–H) and multiple sub-genotypes by means of molecular evolutionary analyses^34–36^. HBV within a given patient can harbor a variety of additional mutations, further increasing the viral diversity. Genetic variation of HBV has been linked to differences in disease severity, which is influenced by a variety of virologic parameters, including differences in replicative fitness and viral protein expression^37^. To better understand this genetic diversity, infections should optimally be established with patient-derived viruses, which has proven difficult.

**Fig. 3:**
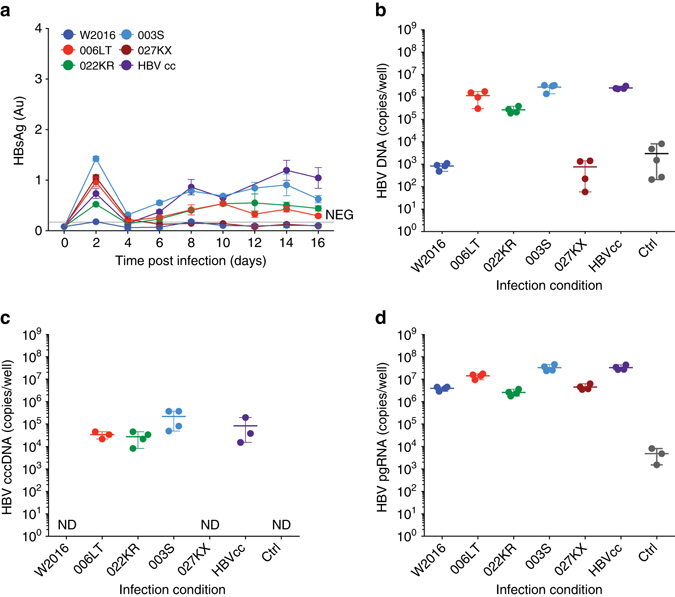
Long-term persistent infection of SACC-PHHs with patient-derived HBV. Assessment of heparin column-purified HBVpat infection of mixed donor HU1008 SACC-PHHs. Quantification of HBsAg concentrations in the supernatants **a**, and total HBV DNA **b**, cccDNA **c**, and pgRNA **d** in SACC-PHH lysates. For all HBVpat infections of mixed donors four biological replicates were performed. All data are presented as means ± s.d

To determine whether infection of SACC-PHHs is limited to HBVcc, we infected the cultures with heparin column-purified, patient-derived HBV (HBVpat) (Supplementary Fig. 6a, b). While exposure of 3B10 cells to HBVpat led to no productive infection (Supplementary Fig. 6c–f), HBV from 3/5 patients resulted in infection levels similar to those reached with HBVcc as assessed by HBsAg enzyme-linked immunosorbent assay (ELISA) (Fig. 3a). Consistent with the rise in HBsAg levels, HBV DNA (Fig. 3b), cccDNA (Fig. 3c, Supplementary Fig. 3 **)** and pgRNA (Fig. 3d) were higher in SACC-PHHs as compared to challenged 3B10 cells and non-infected controls (Supplementary Fig. 6c–f).

### HBV infection in microscale hepatocyte culture formats

**Fig. 4:**
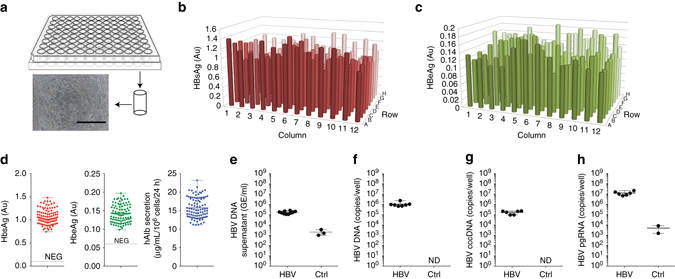
Robust HBV infection SACC-PHHs in microwell formats. **a** Schematic depiction and representative bright-field image of the microwell SACC-PHH system (scale bar = 400 μm). Quantification of HBsAg **b** and HBeAg **c** across the 96-well format at 10 dpi (day 20 post seeding). **d** Limited variation of HBsAg (*left*, mean 1.070 Au, std 0.167 Au, two tail *t*-test *p*-value < 0.0001, compared to 1 Au), HBeAg (middle, mean 0.142 Au, std 0.024 Au, two tail *t*-test *p*-value < 0.0001, compared to 0 Au) and hAlb (right, mean 15.75 μgml per 10^6^ cells per 24 h, std 2.679 μgml per 10^6^ cells per 24 h, two tail *t*-test *p*-value < 0.001, compared to 15 μgml per10^6^ cells per 24 h) at 10 dpi. Quantification of HBV DNA in culture supernatants **e** at 10 dpi, and total HBV DNA **f**, cccDNA **g** and pgRNA **h** in cell lysates of randomly picked wells at 30 dpi. For HBV DNA and RNA quantifications six to ten replicates were performed. For HBsAg, HBeAg, and hAlb 96-biological replicates were performed. All data are presented as means ± s.d

Having established robust and persistent HBV infections in a 24-well format, we then aimed to determine whether the platform could be scaled to a format amenable to high throughput screening (HTS) applications. SACC-PHHs prepared with hepatocytes from a pooled donor were established in 96-microwell plates (Fig. 4a) and exposed to HBVcc 10 days following infection, HBsAg, HBeAg and human albumin, with the latter serving as a marker of cellular functionality, were secreted uniformly with minimal variation (coefficient of variation for HBsAg = 15.57%, HBeAg = 16.61%, human albumin (hAlb) = 17.58%) across the entire plate (Fig. 4b–d). As further evidence for a productive HBV infection, we quantified HBV DNA in supernatants (Fig. 4e) and cell lysates (Fig. 4f), along with cccDNA (Fig. 4g), and pgRNA (Fig. 4h) in wells randomly chosen across the plate. Increases in all these parameters demonstrated that SACC-PHHs in the HTS format were robustly infected.

### Inhibition of viral entry using HBV preS1-derived peptides

**Fig. 5:**
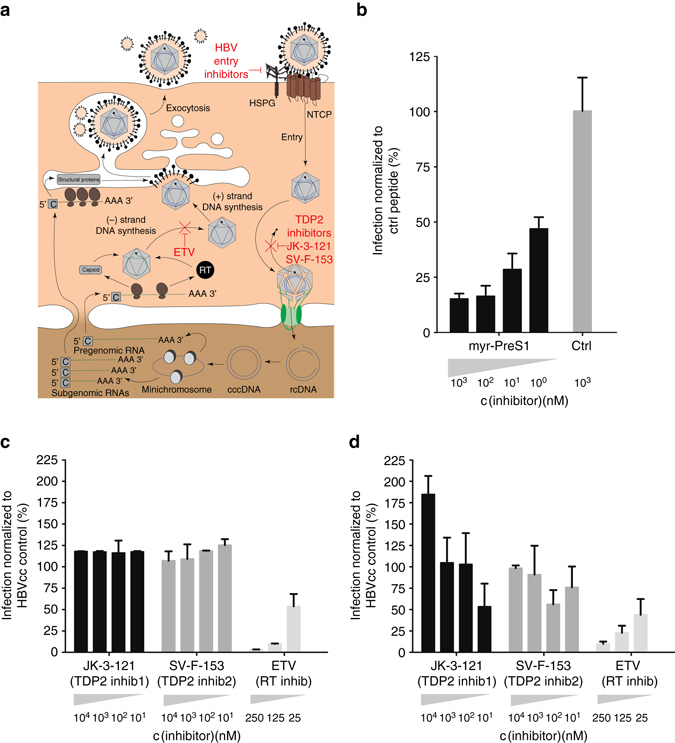
Utility of SACC-PHHs for antiviral drug testing. **a** Schematic of HBV life cycle indicating the presumed mechanism of action of myr-PreS1 entry inhibitor, TDP2 inhibitors and ETV. **b** Prophylactic treatment with myr-preS1-derived peptides in SACC-PHH 96-well format (HU1007 mixed donor). Prophylactic **c** and therapeutic **d** drug dosing of SACC-PHHs (mixed donor HU1008) in 96-well format for nucleotide analog ETV and TDP2 inhibitors (JK-3-121, SV-F-153), *x*-axis: concentration of different drugs. *y*-axis: amount of HBsAg secretion normalized to that secreted by HBVcc infected untreated control cells. For HBVcc infections and drug treatments, six biological replicates were performed. All data are presented as means ± s.d

HBV utilizes the bile-acid transporter NTCP to enter hepatocytes (Fig. 5a)^1, 2^ This process is—in part—facilitated through the physical interaction of the myristoylated first 48 amino acids of the large HBsAg with amino acids 157–165 of NTCP^38^. It was previously shown that acylated peptides derived from the large HBsAg can block virus entry in vitro^25^ and in human liver chimeric mouse models^26^. Here, we aimed to establish proof-of-concept for the utility of the SACC-PHH platform to test such entry inhibitors. HBVcc was pre-incubated with different amounts of HBV preS/2–48^myr^ or a control peptide in which the NTCP binding domain was substituted with a quintuple alanine sequence in positions 10–14, abolishing the ability of the peptide to inhibit HBV infection^38^. Following infection and removal of the inoculum, peptides were added at the appropriate concentrations to the media. In line with previous studies, the HBV preS1-derived peptide efficiently blocked HBV infection in the SACC-PHH cultures, whereas the control peptide had no effect (Fig. 5b).

### Pharmacologic TDP2 inhibition does not suppress HBV viremia

Next, we tested the relative efficacy of various direct-acting antivirals (DAAs) and putative host-targeting antivirals (HTAs) to suppress HBV viremia in the HTS format. Entecavir (ETV, Baraclude) is an inhibitor of HBV reverse transcriptase (RT) that is widely used in clinical practice (Fig. 5a). Recent biochemical data implicated TDP2 as a candidate enzyme for removal of the RT from rcDNA in a step crucial for cccDNA formation (Fig. 5a **)** ^27^. However, a follow up in vitro study showed only a minor impact of knock-down or knock-out of TDP2 on cccDNA formation in hepatoma cells, suggesting other redundant enzymes are likely involved in catalyzing RT removal^39^. To test the effect of pharmacologic inhibition on HBV infection, we utilized two small molecules (JK-3-121 and SV-F-153, Supplementary Fig. 7a) previously shown to efficiently suppress the activity of human TDP2 with high selectivity^40^. To corroborate that JK-3-121 and SV-F-153 indeed had an inhibitory effect on this enzyme, we produced recombinant human TDP2 in *E. coli* (Supplementary Fig. 7b). Following established protocols, we obtained high yields of hTDP2, which was purified by nickel affinity column size exclusion chromatography (Supplementary Fig. 7c) ^40^. hTDP2 has Mg^2+^-dependent activity on 5′-phosphotyrosylated (5′-Y) termini of single-stranded DNA or on duplex substrates with 5′ overhangs of one to four nucleotides and is thought to be involved in the removal of the viral polymerase from HBV rcDNA (Supplementary Fig. 7d **)**. To validate that JK-3-121 and SV-F-153 effectively inhibit the enzymatic activity of TDP2, we employed an assay reported for TDP2 activity on a synthetic substrate, in which methylumbelliferon (MU), a Tyr mimic, is attached via a phosphodiester bond to the 5′-end of a 14-mer DNA oligo corresponding to the 5′-end of duck HBV (-) strand DNA (MUP-DNA) (Supplementary Fig. 7e) ^27^. Incubation of TDP2 with MUP-DNA results in cleavage of the phosphotyrosyl bond, releasing fluorescent MU from non-fluorescent MUP-DNA, which can be monitored in real time using a fluorescence reader. Addition of JK-3-121 and SV-F-153 effectively inhibit this process, thus confirming the efficacy of these compounds (Supplementary Fig. 7f) ^40^.

ETV administration led to a reduction in HBsAg secretion in a dose-dependent fashion, both when supplied prophylactically, i.e., prior to infection (Fig. 5c; Supplementary Fig. 8a), or therapeutically (Fig. 5d). In contrast, inhibition of TDP2 had no effect on HBV infection in either setting (Fig. 5c, d; Supplementary Fig. 8b, c). Of note, results from equivalent drug dosing experiments in 3B10 cells were considerably more variable (Supplementary Fig. 8d, e), showing the utility of SACC-PHHs over the current system. The SACC-PHHs remained healthy, as indicated by hAlb levels, in both pharmacological inhibition settings (Supplementary Fig. 8f, g).

## Discussion

Study of human hepatotropic pathogens has been historically difficult due to the scarcity of experimental models. HBV is a prime example of such a pathogen, which almost exclusively infects human hepatocytes. Numerous attempts have been undertaken to establish HBV infection in PHHs with highly varying efficiency (reviewed in ref. ^17^), with none successful in microscale formats. Fetal human hepatocytes (FHHs) and human hepatocyte-like cells (HLCs) derived from induced pluripotent stem cells (iPSCs) have also been shown to be susceptible to HBV infection, but neither exhibits the fully mature adult PHH phenotype^14, 16, 41, 42^, limiting their utility for antiviral drug screening. Furthermore, while HLCs can be produced in unlimited quantities through directed differentiation of iPSCs, few FHHs can be isolated from a single donor, increasing inter-experimental variability. Hepatocyte monocultures remain the gold standard, but in most studies, viral infections are abortive due to the rapid loss of hepatic functions^13, 15^. More recent efforts have focused on sophisticated tissue engineering approaches employing co-cultures of PHHs and non-parenchymal cells to stabilize the hepatic phenotype^43, 44^. In a previous study, HBV was able to infect PHHs in MPCCs, but infection required JAK inhibition, was limited to specific hepatocyte lots and ceased within 14–19 days post infection^22^. While the severity of chronic hepatitis B has been linked to candidate alleles in genome-wide association studies, there is no evidence for widespread resistance to HBV infection, and thus almost any high-quality hepatocyte donor lot should be permissive to HBV infection^45, 46^. Furthermore, HBV is considered a “stealth” virus that does not strongly induce cell-intrinsic antiviral defense pathways. Based on infection of chimpanzees and humanized mice, it should not be necessary to suppress antiviral signaling^47^. The SACC-PHHs described here enable bona fide HBV persistence beyond 30 days of infection.

Here, we demonstrated that cells in this culture format are susceptible to HBVcc. Between SACCs generated with pooled or single donors, we observed minor differences in the quantity of cccDNA and pgRNA (Figs 1d, e and 2c, d). While hepatocytes from each donor are mixed in equivalent proportions in the pooled lots, this may not necessarily translate to equal representation of each donor after plating. It is also conceivable that some of the donors in the pooled lots are less susceptible to HBV or the highly susceptible donors are underrepresented due to the above-mentioned differences in plating efficiency. In combination, these factors may explain the differences in the quantities of certain HBV replication intermediates. SACC-PHHs can also be infected with patient-derived viruses, which opens the possibility of studying genetically diverse viruses. Three out of five of the HBVpat samples led to robust, persistent infection while the remaining two failed to infect either SACC-PHHs or 3B10 cells. This is likely due to varying numbers of infectious particles in the preparation and the presence of neutralizing antibodies. Co-culture of PHHs with the stromal cells stabilized the hepatic phenotype, which was ultimately responsible for the high permissiveness to HBV. In contrast, our experimental hepatocyte monocultures rapidly deteriorated within 8 to 9 days post seeding and were consequently insusceptible or only minimally susceptible to HBV, even when exposed to the virus shortly after seeding. In contrast, robust infection could be established in the SAC-PHH format as late as 15 days following plating, giving greater flexibility in experimental design, e.g., for genetic manipulations prior to viral infection.

We also established proof-of-concept for the utility of this platform for antiviral drug testing using both DAAs and HTAs. Inhibition of HBV glycoprotein-mediated entry is being pursued as a therapeutic approach to prevent HBV infection, such as after liver transplantation, and might also restrain virus spread in chronically infected patients. In line with previous results obtained in HBV permissive lines and human liver chimeric mice^1, 9, 48–50^, we validated that a preS1-derived peptide can efficiently interfere with HBV uptake into PHHs in the SACC platform. Administration of the candidate entry inhibitor, Myrcludex B, which is a preS1-derived peptide, has been shown to efficiently deplete nuclear cccDNA in humanized mice by preventing HBV reinfection^50^. Blocking viral entry with Myrcludex B can also suppress HDV, a small viroid that hijacks the HBV envelope, in patients persistently infected with HBV and HDV^51, 52^. Thus, the SACC-PHH platform may have utility for assessing preclinically the efficacy of other entry inhibitors and possibly (vaccine-induced) neutralizing antibodies.

Inhibitors of the HBV RT are widely used to suppress HBV viremia in patients and also lead to a dose-dependent reduction in HBV infection in the SACC-PHHs in the 96-microwell format. To achieve a (functional) cure for chronic hepatitis B, elimination or permanent inactivation of cccDNA remains a priority. Biochemical data implicated TDP2 in the removal of the covalently attached RT from the incoming rcDNA, a crucial step preceding the formation of cccDNA^27^. However, genetic disruption of TDP2 did not lead to a reduction in HBV viremia^39^, suggesting redundancy in the presumed host enzymes that can facilitate RT removal. We provide evidence that pharmacologic inhibition of TDP2 is insufficient to suppress HBV viremia, which is consistent with previous genetic studies^39^. Numerous alternative approaches are being pursued targeting HBV cccDNA, and the SACC-PHH platform presented here may aid in the identification and testing of novel therapeutic regimens.

## Methods

### Cell lines

In all, 293 T (American Tissue Culture Collection, ATCC® Number: CRL-3216^TM^, Manassas, VA) and HepG2 cells (American Tissue Culture Collection, ATCC® Number: HB-8065™, Manassas, VA) were maintained in Dulbecco’s modified Eagle medium (DMEM; ThermoFischer, Waltham, MA) base medium supplemented with 10% (vol/vol) fetal bovine serum. HepG2.2.15 cells^7^ (kindly provided by Dr Christoph Seeger, Fox Chase Center) were maintained in DMEM/10% FBS media containing tetracycline (Sigma Aldrich, St Louis, MO) at 10  μg/ml. 293 T cells were grown on tissue culture-treated plastic ware (Corning Inc., Corning, NY) and HepG2 and HepG2.2.15 cells on type IV collagen-coated plates (Sigma Aldrich, St Louis, MO).

### Generation of human NTCP expressing HepG2 Cells

The hNTCP-eGFP lentiviral vector was generated as follows. A hNTCP-eGFP fusion protein was created by overlap PCR. In the first round of PCR: Round 1, Reaction 1: hNTCP was amplified from pCMV-Sport6 Slc10A1 hNTCP (OpenBiosystems, now Dharmacon, Lafayette CO) with primers introducing flanking 5′ XbaI site, 5′ Kozak sequence and 5′ FLAG to the hNTCP ORF, as well as a 3′ linker sequence (GGCAGC) and overlap fragment from the eGFP coding sequence. Round 1, Reaction 2: eGFP was PCR amplified from pShuttleCMV-eGFP (Addgene catalog #16403) (previously functionally characterized version of eGFP) to introduce 5′ overhang of overlapping end of hNTCP ORF and linker sequence, and 3′ *Xho*I site. In the second round of overlap PCR, the two products from the first round of PCR (modified hNTCP and modified eGFP) were use as template with the external primers to yield the final PCR product: XbaI-KOZAK-FLAG-hNTCP(GGCAGClinker)eGFP-*Xho*I. This PCR product as well as the backbone vector pTrip^53^ were digested separately with *Xba*I/*Xho*I and resulting backbone and insert were ligated together to form the final construct.

Next HepG2 cells were transduced with a hNTCP-eGFP lentivirus. Lentivirus was generated by Xtremegene (Roche Applied Science, Indianapolis, IN) mediated co-transfection of 293 T cells with plasmids encoding (1) a minimal HIV pTRIP with hNTCP-eGFP transgene, (2) gag-pol from HIV^53^ and (3) appropriate viral glycoproteins (VSV-G)^53^. Pseudoparticle-containing supernatants were harvested at 24 and 48 h, pooled and filtered (0.45 μm pore size Millipore, Darmstadt, Germany). Pseudoparticle infections were performed in the presence of 4 μg/ml polybrene. After 3 days cells were then single cell sorted using a Bio-Rad S3 Cell Sorter (Bio-Rad, Hercules, CA) into a collagen coated 96-well plate. Cells were expanded and assessed for hNTCP-eGFP expression using a LSRII Multi-Laser Analyzer (BD, Franklin Lakes NJ) at the Princeton flow cytometry core facility.

### HBcAg FACS assay

To assess the susceptibility of hNTCP-eGFP HepG2 clones to HBV infection, cells that had been challenged with HBV were first trypsinized and then fixed with FACS fixation buffer (1% PBS, 1% PFA) for 20 min at RT. After fixation, cells were centrifuged and re-suspended in permeabilization buffer (1% FBS, 0.1% saponin, in 1× PBS) for 20 min at RT. After permeabilization, cells were again centrifuged and were re-suspended with 50 ul of HBcAg primary antibody (1:200 diluted in permeabilization buffer; HBcAg goat-anti-mouse (Fisher Scientific, cat# MA7609 Waltham, MA) and incubated for 30 min at 4 °C. After the incubation cells were washed twice with FACS Buffer (1× PBS, 1% FBS). Cells were then re-suspended and incubated with an Alexa 647 anti-mouse secondary antibody (1:250 dilution in permeabilization buffer; Fisher Scientific, Waltham, MA) for 30 min at 4 °C. Cells were then washed twice with FACS buffer to remove any excess secondary antibody and were then run on a LSRII Multi-Laser Analyzer (BD, Franklin Lakes, NJ) at the Princeton flow cytometry core facility.

### Generation of self-assembling primary hepatocyte co-cultures (SACC-PHHs)

Cryopreserved human hepatocytes were obtained from Bioreclamation IVT Inc. (Westbury, NY) [Hurel lot ID = vender ID: Hu1003 = JMG, Hu1007 = YMD, Hu1010 = TLQ], ThermoFisher Scientific (Waltham, MA) [Hu1004 = HU1552], Sekisui Xenotech LLC (Kansas City, KS) [Hu1008 = 1410235], and Corning Inc. (Corning, NY) [Hu1002 = BD304, Hu1013 = BD317].

The co-culture model consists of a mixture of human hepatocytes and non-parenchymal mouse embryonic fibroblast 3T3-J2 cells (American Tissue Culture Collection, ATCC® Number: CCL-92^TM^, Manassas, VA)^19, 28–30^. Cryopreserved hepatocytes were removed from liquid nitrogen and thawed in a water bath at 37 °C. Hepatocytes were transferred to a 50 ml conical tube containing 20 ml plating medium (Hµrel PlatinumHeps plating medium™, Hurel Corporation, New Brunswick, NJ), and centrifuged at 150x*g* for 10 min at room temperature. After removing the supernatant, the cells were re-suspended in Hµrel Platinum Heps plating medium™ and cell number as well as cell viability were assessed using trypan blue exclusion. Mouse embryonic fibroblast 3T3-J2 cells were cultured in DMEM (Inoza, Walkersville, MD) supplemented with 10% heat-inactivated fetal bovine serum, 200 U/ml penicillin/streptomycin. Cells were maintained at 37 °C in a 5% CO_2_: 95% air-humidified atmosphere until used for experimental plating. On plating day, cells were detached from the plate surface using trypsin (0.25%), suspended in 15 ml DMEM medium and centrifuged at 200x*g* for 5 min at room temperature. After removing the supernatant, the cells were re-suspended in plating medium (Hµrel PlatinumHeps™, Hurel Corporation, New Brunswick, NJ) and cell number and viability were determined using trypan blue exclusion.

All co-cultures were plated on collagen type-I coated, tissue culture treated plates 96-well and 24 well (Corning Inc, Corning NY). Hepatocytes were seeded at a seeding density of 30,000 and 188,000 hepatocytes in each well of a 96-well and 24-well plate, respectively. 3T3-J2 cells were added the next day at 15,000 and 90,000 in each well of well of a 96-well and 24-well plate, respectively. Hurelhuman™-24 and Hurelhuman™**-**96 SACC-PHH are distributed by the Hurel Corporation (New Brunswick, NJ). Cells were maintained in 500 μl for 24-well plates and 150 μl for 96-well plates, in Hµrel PlatinumHeps maintenance medium™, (Hurel Corporation, New Brunswick, NJ). Medium was replaced every 2 days. The cells were co-cultured at 37 °C in a 5% CO_2_ for 10 days prior to HBV infections.

### Characterization of hepatocyte function

All experiments were performed in 96-well tissue culture treated plates with a compound incubation volume of 100 μl. On the day of the experiment, cultures were incubated with 5 μM of midazolam, 20 µM dextromethorphan, or 20 μM of tolbutamide (Sigma, Missouri, USA) prepared in dosing medium (Hurel Corporation, New Brunswick, NJ) at 37 °C and 5% CO_2_. Incubations were stopped after 1 h and metabolite formation was monitored. Metabolites were assayed for 1-OH midazolam, dextrorphan, and 4-OH tolbutamide. These are indicative of CYP3A4, CYP2D6, and CYP2C9, respectively. The experiment was terminated by removing 100 μl of supernatants which were immediately frozen at −20 °C.

Formation of metabolites was measured using liquid chromatography–mass spectrometry (LC-MS)/MS at Hurel’s facilities (New Brunswick, NJ). Samples were centrifuged at 500x*g* for 10 min before injecting 10 μl of each sample. The LC-MS/MS system comprised a Shimadzu LC-10ADvp pump (Shimadzu, Columbia, MD), SIL-HTS autosampler (Shimadzu, Columbia, MD), and an API 4000 mass spectrometer with a Turbo Ion Spray probe (Applied Biosystems/MDS SCIEX, Ontario, Canada). The separation of compounds was achieved using a reversed stationary phase (Advantage ARMOR C-18, 5 mm, 30.0-2.1 mm, Analytical Sales and Services, Inc., Pompton Plains, NJ). A fast gradient using mobile phases of 0.1% formic acid in acetonitrile and water with 0.1% formic acid along with switching valves and pumps was used for analysis. Phenomenex C18 Synergi 50 × 2.00 mm was used as the analytical column.

Collected medium samples were analyzed for urea concentrations, via a modified Berthelot reaction where phenol is replaced by salicylic acid, using a commercially available assay kit (Stanbio Enzymatic Urea Nitrogen (BUN) Procedure no. 2050) scaled down for use in a 96-well plate. Absorbance was read in a Tecan Ultra 384 microplate reader at 600 nm.

### Production of cell-culture-derived HBV

HepG2.2.15 cells^7^ were grown in media containing tetracycline until they reached a confluency of 100%. At this time media was changed to DMEM F12 media supplemented with 10% FBS, 1% Pen/Strep. Media from the HepG2.2.15 culture was collected every 2 to 3 days for approximately 4 weeks. The collected media was sterile filtered through a 0.22 μm filter (Millipore, Darmstadt, Germany) and was then concentrated *ca*. 100-fold using a stir cell concentrator (Millipore, Darmstadt, Germany). After concentration, the virus was run over a 5 mL HiTrap heparin column (GE, Fairfield, CA) to further concentrate and purify infectious virus particles from non-infectious sub-viral particles. Concentrated virus was applied to a heparin column, which was washed with 5-column volumes of wash buffer (20 mM phosphate buffer, 50 mM NaCl, pH = ~ 7). Afterwards, the virus was eluted with elution buffer (20 mM phosphate buffer, 2 M NaCl, pH = ~ 7). Once all virus was eluted, the viral stock was dialyzed using a dialysis cassette (Millipore, Darmstadt, Germany). After dialysis, virus was aliquoted into cryovial tubes and cryopreserved at −80 °C until use.

### Purification of HBV from patient plasma samples

Cryopreserved plasma samples from de-identified, chronic HBV carriers were kindly provided by Susan Stramer (American Red Cross, Gaithersburg, MD). To purify infectious HBV virions and remove coagulation factors the patient plasma samples were loaded onto a 1 ml HiTrap heparin column (GE, Fairfield, CA). The samples once loaded were then washed with 2-column volumes worth of wash buffer (20 mM phosphate buffer, 50 mM NaCl, pH = ~ 7). This helped aid in the removal of non-infectious sub-viral particles as well as remove some of the coagulation factors present in the patient plasma. The infectious virus was then eluted using elution buffer (20 mM phosphate buffer, 2 M NaCl, pH = ~ 7). Once the purified infectious HBV was isolated it was dialyzed using a 3 ml dialysis cassette (Millipore, Darmstadt Germany) in sterile 1× PBS. After dialysis, virus was stored at 4 °C until use 24 h later.

### HBV infections

HBV infections of SACC-PHHs, HepG2 overexpressing hNTCP, and un-modified HepG2 cells were performed as follows. HBV infections with tissue culture-derived HBV from HepG2.2.15 cells was used at a MOI of 4000 unless indicated otherwise, in the presence of 4% polyethylene glycol (PEG) 8000 (Sigma-Aldrich, St Louis, MO) 0.5% dimethylsulfoxide (DMSO, Sigma-Aldrich, St. Louis, MO).

For HBV infections with purified patient plasma samples HepG2 cells overexpressing hNTCP were challenged with either 7.5, 15, or 30% (v/v) patient plasma per well. SACC-PHHs were challenged with 30% (v/v) patient plasma per well. Regardless, for the infections, the medium of all cells was supplemented with MgCl_2_ (6 mM), and CaCl_2_ (9 mM), and 100 μM heparin in order to prevent coagulation.

For monoculture infections of HU1003 the same viral stock of tissue-derived HBVcc was used as for the SACC-PHH HU1003 for direct comparison purposes. The monocultures were plated and the following day a pre-treatment with 0.5% DMSO was started. On day 2, post plating the cells were challenged with an MOI of 4000 virions for 18 h. After, challenge the inoculum was removed by washing the cells five times with maintenance media. Fresh maintenance media containing 0.5% DMSO was added. The media was changed every 2 days. Cells were harvested on day 8 post HBV infection.

### Human albumin ELISA

Chromatographically purified human albumin was obtained from MP Biomedicals (catalogue #2191349, Santa Ana, CA) and horseradish peroxidase-conjugated goat IgG to human albumin was obtained from Bethyl Labs (catalogue # A80-129P, Montgomery, TX). Following completion of the desired infection and treatment period, serum albumin content was quantified by competitive ELISA. All 96-well and 24-well plates were assayed in an identical manner.

### HBV surface antigen ELISA

Detection and quantification of HBsAg levels was performed by ELISA according to the manufacturer’s instructions (GS HbsAg EIA 3.1, Bio-Rad, Hercules, CA). Briefly, a 100 µl sample of a 1:20 dilution of supernatant was prepared in 1x PBS was used in lieu of undiluted supernatant. Absorbance was read at 450λ on the BertholdTech TriStar (Bad Wildbad, Germany).

### HBV envelope antigen ELISA

Detection and quantification of HbeAg levels was performed by ELISA according to the manufacturer’s instructions (Abnova, Taipei, Taiwan). Briefly, a 100 ul sample of 1:10 diluted supernatant was used in lieu of undiluted supernatant. Absorbance was read at 450λ on the BertholdTech TriStar (Bad Wildbad, Germany).

### HBV DNA isolation from supernatants

HBV DNA was isolated following the Qiamp MinElute Virus Spin Kit (50), (Qiagen, Hilden, Germany). HBV DNA was eluted in 60, and 5 µl was used per well in the HBV DNA quantitative PCR (qPCR) reaction.

### Total HBV DNA isolation and quantification from infected cells

To isolate total HBV DNA from HBV challenged SACC-PHHs, 3B10, or HepG2 cells, 300 µl (24 well) or 100 µl (96-well) of lysis buffer was added, respectively, to the corresponding sample (50 mM Tris-Base, 50 mM EDTA, 1% SDS, 100 mM NaCl pH 8.0). The sample was further digested through the addition of 20 µl of Proteinase K per sample from a QIAMP DNA mini kit (Qiagen, Hilden, Germany) for an hour at 37 °C. After digestion with Proteinase K, 1 µl of Rnase A (Sigma Aldrich, St Louis, MO) was added to the lysate and incubated at room temperature for 2 min. Five-hundred microliters of AL lysis buffer (Qiagen, Hilden, Germany) was subsequently added to the solution. The samples were incubated at 70 °C for 4 h, vortexed every 20 min to digest the cells completely. Following this step, 500 µl of 100% EtOH were added and mixed thoroughly by inverting 10 times. This suspension was then applied to a Qiamp DNA mini kit column and centrifuged for 1 min at 16,000x*g*). The samples were spun again in new tubes for 1 min at 16,000x*g* to dry. The DNA was then eluted with 50 µl of AE buffer and concentrations measured using a Nanodrop spectrophotometer (Thermo Fischer Scientific, Waltham, MA).

A 5 µl aliquot of HBV DNA isolated from lysed cells was used per reaction well. To amplify HBV DNA the following primers and probes were used: CCGTCTGTGCCTTCTCATCTG (forward primer), AGTCCAAGAGTCCTCTTATGTAAGACCTT (reverse primer), and probe FAM-CCGTGTGCACTTCGCTTCACCTCTGC-TAMRA^22^. Primers were kept at a concentration of 600 nM and probe at 300 nM final concentration in the reaction. A master mix was created containing 2× TaqMan reaction mix (Applied Biosystems, Foster City, CA), primer/probe mix and ddH_2_O. The master mix was then applied with the samples to the respective wells. Five microliters of the standards and the samples were added to the respective wells. The following PCR program was run on a Step One Plus qPCR machine (Life Technologies, Carlsbad, CA): 50 °C for 5 min, 95 °C for 10 min, followed by 40 cycles of 95 °C for 15 s, 56 °C for 40 s, and 72 °C for 20 s. Lastly, a melt curve was performed at 95 °C for 10 s, 65 °C for 10 s, 50 °C for 5 s, and 95 °C for 5 s.

### HBV pgRNA isolation and quantification from infected primary hepatocytes

SACC-PHHs were lysed with 350 µl RLT buffer (Qiagen RNAeasy kit, Qiagen, Hilden Germany) supplemented with 2-Mercaptoethanol for 10 min at RT. The cells were then pipetted into an RNase free Eppendorf tube. The cell suspensions were then passed through a 26½ gauge needle five times in order to facilitate cell lysis. Once completed, the manufacturers protocol for the Qiagen RNAeasy kit (Qiagen, Hilden, Germany) was followed except for elution where the sample was eluted twice once with 50 µl of Rnase free water and then addition 30 µl.

To quantify HBV pgRNA a modified iTaq Universal SYBR Green One-Step qPCR kit (BioRad, Hercules, CA) protocol was used. A primer mix with each primer at 3 µM was created with the forward primer GAGTGTGGATTCGCACTCC and the reverse primer GAGGCGAGGGAGTTCTTCT^2^. A master mix was created as follows per reaction: 5 µl of SYBR mix, 0.125 µl of RT, 1 µl of primer mix, and 1.875 µl of ddH_2_O. The following cycling time was used: reverse transcription and amplification step at 50 °C for 10 min and 95 °C for 1 min; 40 cycles of 95 °C 15 s, and 60 °C for 1 min. The melt curve was performed at 95 °C for 5 s, 65 °C for 5 s, 95 °C for 15 s, and 50 °C for 5 s.

### HBV cccDNA isolation and quantification from infected primary hepatocytes

A 25 µl aliquot of the respective total HBV DNA sample isolated from cell lysate was digested with 1 µl plasmid-safe DNAse (Epicentre, E3101K, Madison, WI) to destroy all chromosomal DNA along with any linear form of HBV DNA. According to the manufacturer’s instructions, the reaction mix was incubated at 37 °C for 30 min to 1 h in order to digest the samples. Following the digestion, plasmid-safe DNase was heat inactivated by incubating the samples at 70 °C for 30 min. The cccDNA was then purified using a DNA clean up and concentration kit (Zymo, Irvine, CA), eluted in 30 µl of sterile ddH_2_O, and the residual amount of DNA quantified using a Nanodrop spectrophotometer. This resulted in a 1–1.5 log drop in overall DNA concentration. Samples were either used immediately for HBV cccDNA quantification by qPCR or were stored at −20 °C.

Quantitation of HBV cccDNA was performed as follows. A 5 µl aliquot of HBV DNA either isolated from mouse serum or from liver DNA was used per reaction well. We used the following primers and probes: GTCTGTGCCTTCTCATCTGC (forward primer), AGTAACTCCACAGTAGCTCCAAATT (reverse primer), and probe FAM-TTCAAGCCTCCAAGCTGTGCCTTGGGTGGC-TAMRA. The final concentration of primers was 0.9 µM, 0.2 µM probe, and 4% DMSO. The following qPCR cycling was used: 95 °C for 10 min, followed by 50 cycles of 95 °C for 15 s, and 61 °C for 1 min.

### Immunofluorescence imaging of HbcAg in infected primary hepatocytes

HBV infected SACC-PHHs were washed once with sterile 1× phosphate-buffered saline (PBS, Life technologies, Carlsbad, CA) and then fixed for 20 min with 4% (w/v) paraformaldehyde (PFA, Sigma-Aldrich, St. Louis, MO) at room temperature (RT). After fixation, the cells were washed three times with sterile 1× PBS. Cells were then incubated for 30 min in blocking buffer (10% bovine serum albumin in sterile 1× PBS, Sigma-Aldrich, St Louis, MO). After 30 min the primary anti-HBcAg antibody (DAKO rabbit polyclonal, catalog #B0586, Santa Clara, CA) was added at a 1:1600 dilution for 1 h. After the 1 h incubation with the primary antibody the cells were washed five times with 1× PBS. The secondary was then added (Anti-rabbit-Alexa555 1:1000, Fisher Scientific, Waltham, MA) along with the nuclear Hoechst dye (1:10,000, Sigma-Aldrich, St Louis, MO and the cells were incubated for an additional hour. After incubation, the cells were washed five times with 1× PBS and then had 300 μl of sterile 1× PBS added and the cells were stored at 4 °C until imaging.

### Cloning, expression, and purification of hTDP2

The coding sequence of human TDP2 was PCR amplified from a hTDP2 expression plasmid (NM_016614.2) with Q5 (NEB, Ipswich, MA) and inserted into a pQLinkH (Addgene, plasmid #13667) expression plasmid via restriction digest with *Bam*HI and *Hin*dIII and ligated ON at 16 °C. The sequence of the *N*-terminally 7x His-tagged TDP2 was confirmed by DNA sequencing. BL21-DE3 Rosetta cells (EMD Millipore, Billerica, MA) were transfected with the TDP2 expression plasmid and grown in LB medium at 37 °C until reaching an OD600 of 0.6. Protein expression was induced by the addition of 1 mM IPTG (Sigma-Aldrich, St Louis, MO) for 16 h at 30 °C. The cells were collected via centrifugation and frozen at −20 °C until purified. To purify the TDP2, the pellet was defrosted and re-suspended in 50 ml native binding buffer (500 M NaCl and 50 mM NaH_2_PO_4_, pH 8.0) with 100 mg lysozyme and incubated on ice for 30 min. Cells were sonicated with 6 × 10 s bursts at high intensity with cooling in between. DNase I (Worthington, Columbus, OH) was added at 5 μg/ml and incubated on ice for 15 min. Lysate was spun at 3000x*g* for 15 min and supernatant transferred to clean tube. In all, 1.5 mL Ni NTA resin (Life Technologies, Carlsbad, CA) was transferred to 15 ml column and equilibrated with native binding buffer. Supernatant was run over column twice, and resin was washed 3 × 10 ml with native wash buffer (native binding buffer with 20 mM imidazole). Resin was eluted with 250 mM imidazole in native binding buffer and collected in 1.5 ml fractions. Fractions were analyzed by Sodium dodecyl sulfate polyacrylamide gel electrophoresis (10% acrylamide gel) followed by Coomassie staining. In addition, western blotting was performed using an anti-His antibody (Millipore, clone HIS.H8, catalog #05-949, Billerica, MA) at 1:1000 dilution and visualized with a LiCor Odyssey. Pure fractions were combined and dialyzed twice against native binding buffer using a 10 kDa dialysis cassette (Pierce, Waltham MA) before being further purified via size exclusion chromatography using an S75 16/60 column. Pure protein was then lyophilized and stored at −80 °C until further use.

### In vitro fluorescence hTDP2 inhibition assay

To test the activity of hTDP2 and to corroborate the inhibitory effect of JK-3-121 and SV-F-153 hTDP2 inhibitors, we performed a fluorescence based assay as previously described^40^. First, a 5′-methylumbelliferon (MUP)—phosphodiester bond 5′-GTAATTCTTAAGTTG-3′ oligo was synthesized (IDT, San Jose, CA). The MUP- GTAATTCTTAAGTTG and lyophilized hTDP2 was solubilized in substrate buffer (50 mM Tris-HCl, 80 mM KCl, 2 mM EDTA, 1 mM DTT, 40 ug/mL BSA, 10% DMSO, 5 mM MgCl_2_, pH = 8). For each reaction hTDP2 was at a final concentration of 100 nM, and the MUP-GTAATTCTTAAGTTG oligo was at a concentration of 0.5 μM, in a total reaction volume of 200 μl of substrate buffer. The JK-3-121 and SV-F-153 inhibitors were added to the respective wells at 10, 1, 100, and 10 nM, respectively, and incubated for 30 min at room temperature. A control condition of hTDP2 was used without either inhibitor being present. All conditions were run in quadruplicate, in a black μClear 96-well plate (Greiner-bio-One, Kremsmünster, Austria), in a SpectraMAX 96-well plate reader (Molecular Devices, Sunnyvale, CA) with excitation = 355 nM, and emission = 460 nM.

### Generation of PreS1-fluorescein isothiocyanate (FITC) and inactive PreS1 peptides

The PreS1-FITC peptide (Myr-GTNLSVPNPLGFFPDHQLDPAFGANSNNPDWDFNPNKDHWPEANQVGK-FITC) was purchased from (Life Tein, Somerset, NJ). The inactive PreS1 peptide variant containing a quintuplet alanine amino acids at positions 10–14 was synthesized using Fmoc solid phase synthesis. To avoid aspartimide formation the Fmoc deprotection was carried out with 20% Piperidine in dimethylformamide (DMF) and 0.1 M HOBT at room temperature. Amino acid coupling was performed at 50 °C using baseless DIC/Oxyma double coupling. Myristic acid was coupled to the *N*-terminus for 18 h at room temperature using 10 equiv. Myristic acid, 10 equiv. PyAOP and 20 equiv. DIEA in DMF. The crude peptide was cleaved from the resin (95% trifluoroacetic acid (TFA), 2.5% TIS, and 2.5% H_2_O), precipitated with diethylether and purified using preparative high-performance liquid chromatography (0–95% MeCN in 60 min). The identity of the peptide was confirmed by mass spectrometry: Expected 5229.54 Da, Found = 5229.84 Da. The control peptide sequence was as follows Myr-GQNLSTSNPAAAAADHQLDPAFRANTANPDWDFNPNKDTWPDANKVG-CONH_2_.

### PreS1 prophylactic inhibition assay

Plated SACC-PHHs (mixed donor HU1007) were first incubated for 2 h with either a PreS1-derived peptide conjugated to a FITC label or with an inactive PreS1 variant that contains a quintuplet alanine amino acids at positions 10–14. After the 2 h incubation, the cells were then challenged with HBV (MOI = 4000) in the presence of 0.5% DMSO, and 4% PEG 8000 for 18 h. At the conclusion of 18 h, the cells were then washed five times with media and fresh maintenance media contain 0.5% DMSO was then added with the corresponding concentration of PreS1 or control peptide (1 μM, 100 nM, 10 nM, and 1 nM). Every 2 days, the media was replaced with maintenance media containing 0.5% DMSO and the corresponding concentration of peptide. Supernatants were run for HBsAg ELISA to determine if inhibition of HBV infection had occurred. In addition, a control condition of SACC-PHH’s only challenged with HBVcc without any peptide inhibitor was also performed.

### Drug inhibition assays

Drug treatment with TDP2 inhibitors JK-3-121 and SF-V-153^40^ or with entecavir (ETV, Sigma-Aldrich, St Louis, MO) were performed in 96-well plates seeded with either PHH’s or hNTCP-eGFP HepG2 cells. TDP2 inhibitors were solubilized in 100% DMSO and were stored at −20 °C while ETV was solubilized in sterile 1× PBS. Prior to drug treatment, cells were treated with 0.5% DMSO for 24 h and then were challenged with HBV derived from HepG2.2.15 cells that had been heparin column purified. Once persistent HBV infection was established (day 16) drug treatment was started. A series of concentrations was used for each drug. For TDP2 inhibitors JK-3-121 and SV-F-153 concentrations of 1x10^4^, 1x10^3^, 1x10^2^, and 10 nM, stocks of each TDP2 inhibitor were created in order to maintain the same level of DMSO. ETV was used at final concentrations of 250, 125, and 25 nM. Drug treatment was performed over 18 days during which the inhibitors were freshly supplied every 2 days at each media change with monitoring of HbsAg and hAlb levels over this period of time. At the end of the 18 days, cells were lysed with lysis buffer (50 mM Tris-Base, 50 mM EDTA, 1% SDS, 100 mM NaCl pH 8.0) or with Qiagen RLT supplemented with 2-Mercaptoethanol for 10 min at RT. Total HBV DNA, pgRNA, and cccDNA were quantified. As a control, a set of wells were only challenged with HBV and had no drug treatment administered over the course of the experiment. Ever condition was performed in sextuplets.

Prophylactic drug treatment with TDP2 inhibitors JK-3-121 and SF-V-153 or with ETV was performed in 96-well plates seeded with either PHH’s or hNTCP-eGFP HepG2 cells. TDP2 inhibitors were solubilized in 100% DMSO and were stored at −20 °C while ETV was solubilized in sterile 1× PBS. 1 day prior to HBV challenge cells were treated with respective concentration of either TDP2 inhibitors or ETV. Cells were then challenged with HBV derived from HepG2.2.15 cells that were heparin column purified in the presence of the respective drug. Every 2 days, media was collected and new media with the respective concentration of inhibitor was added in order to maintain a constant level of drug throughout the experiment. For TDP2 inhibitors, JK-3-121 and SV-F-153 concentrations of 1x10^4^, 1x10^3^, 1x10^2^, and 10 nM, stocks of each TDP2 inhibitor were created in order to maintain the same level of DMSO. ETV was used at final concentrations of 250, 125, and 25 nM. Drug treatment was performed over 34 days for SACC-PHHs and for 19 days for hNTCP-eGFP HepG2 cells with monitoring of HbsAg and hAlb levels over this period of time. At the end of the 34 or 19 days, cells were lysed with lysis buffer (50 mM Tris-Base, 50 mM EDTA, 1% SDS, 100 mM NaCl pH 8.0) or with Qiagen RLT supplemented with 2-Mercaptoethanol for 10 min at RT. Total HBV DNA, pgRNA, and cccDNA were quantified. As a control, a set of wells were only challenged with HBV and had no drug treatment administered over the course of the experiment. Ever condition was performed in sextuplets.

### Data availability

The data that support the findings of this study are available from the corresponding author upon request.

## Electronic supplementary material

Supplementary Information
