# Supplementary material for: Long-term hepatitis B infection in a scalable hepatic co-culture system
Source: Nat Commun. 2017 Jul 25;8:125. doi: 10.1038/s41467-017-00200-8 (PMC5527081; doi:10.1038/s41467-017-00200-8)
Supplement: Supplementary file 1 — Supplementary Information [file 41467_2017_200_MOESM1_ESM.pdf]

File name: Supplementary Information
Description: Supplementary Figures and Supplementary Tables

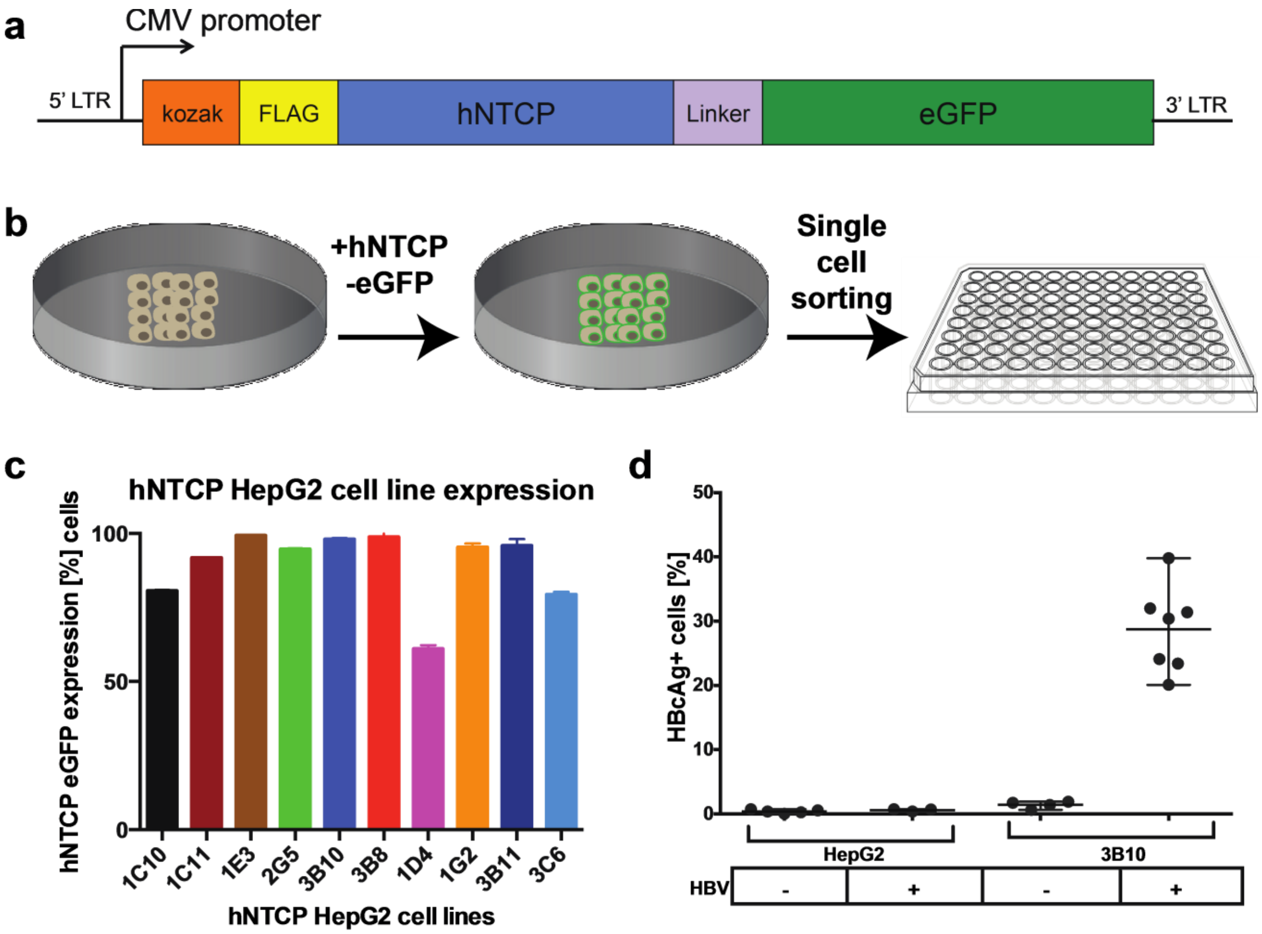

Supplementary Figure 1. Generation and characterization of hNTCP-eGFP expressing HepG2 cells. (**a**) Schematic of hNTCP-eGFP lentiviral construct. (**b**) Process of generating hNTCP-eGFP expressing HepG2 clones. (**c**) Analysis of hNTCP-eGFP expression as assessed by flow cytometric analysis of expanded hNTCP-eGFP clones. (**d**) hNTCP-eGFP clone 3B10 had the greatest susceptibility to HBV infection as assessed by HBcAg staining FACS analysis as compared to HepG2 controls. Three biological replicates were performed for panel (**c**) and four to seven biological replicates were performed for panel (**d**). All data are presented as means ± s.d.

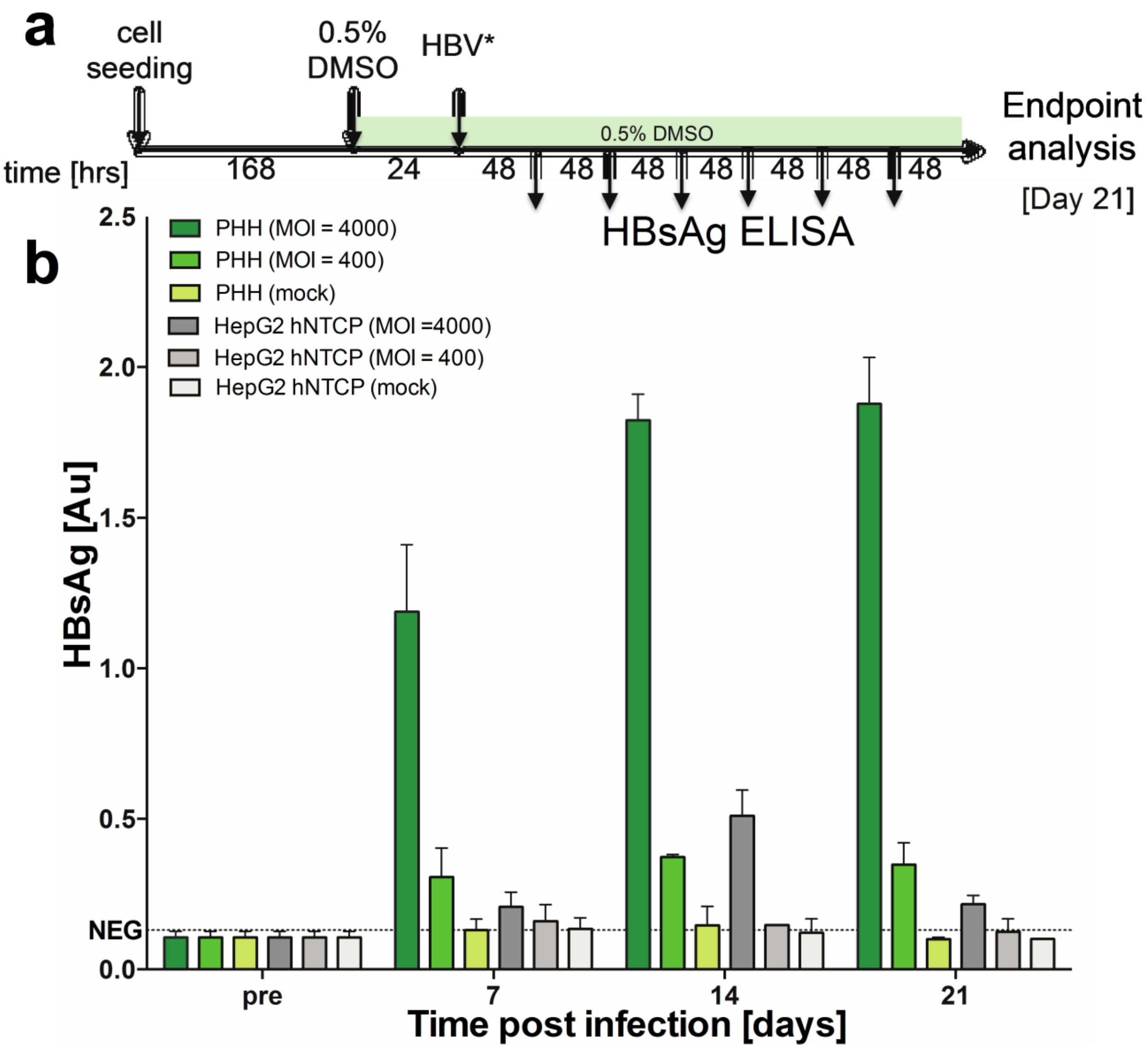

Supplementary Figure 2. Comparison between HBV infection in mixed donor SACC-PHHs and hNTCP-eGFP HepG2 cells over a titration range. (**a**) Schematic representation of the experimental time course. (**b**) Kinetics of an HBV infection in SACC-PHHs and hNTCP-eGFP HepG2 cells as assessed by HBsAg ELISA. Each experimental condition had three biological replicates performed. All data are presented as means ± s.d.

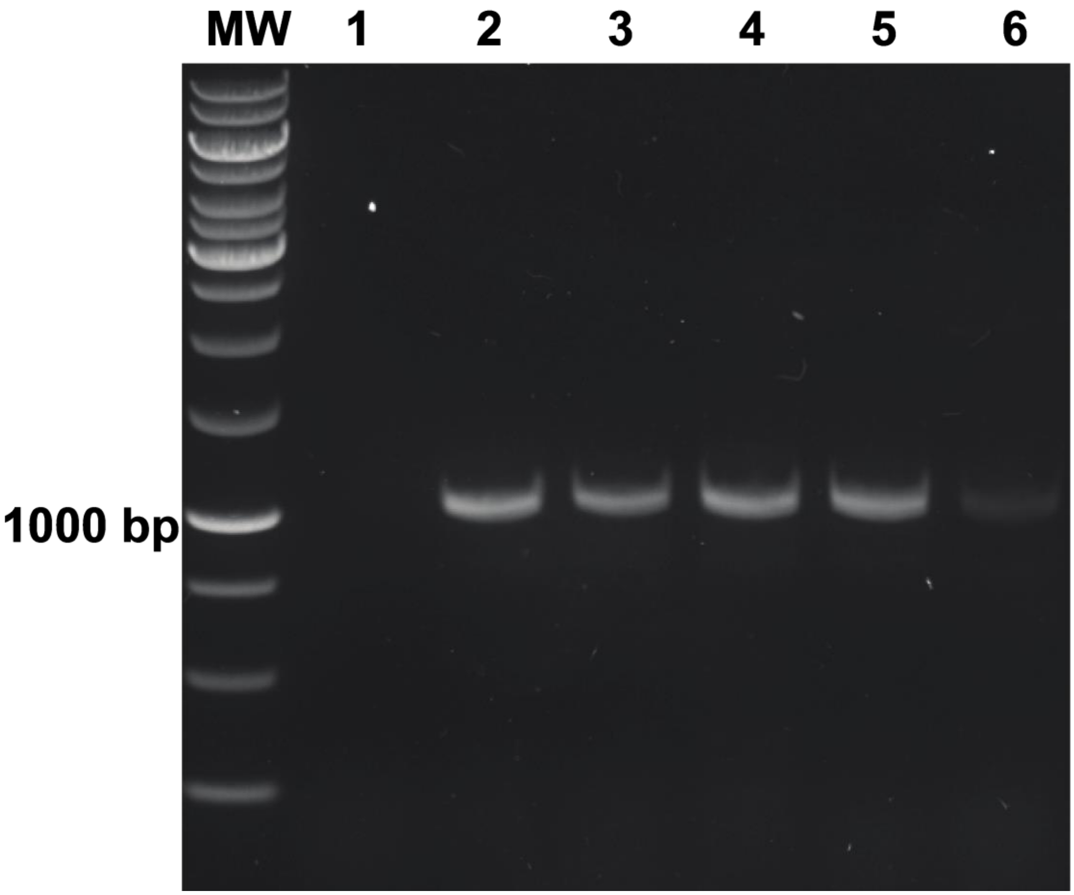

Supplementary Figure 3. Agarose gel analysis of plasmid safe digested HBV cccDNA samples with cccDNA biased primers. Agarose gel analysis of plasmid safe nuclease digested HBV cccDNA samples from cell lysates. Lane 1 = negative control, lane 2 = Single donor HU1004 (day 40 cell lysate), lane 3= HU1008 mixed donor sample (day 40 cell lysate), lane 4= HU1007 mixed donor sample (day 32 cell lysate), lane 5= SACC-PHH HBVpat 003S challenged, cell lysate (Day 16), lane 6= HBVcc no drug treatment SACC-PHH HU1008 96 well cell lysate (Day 34).

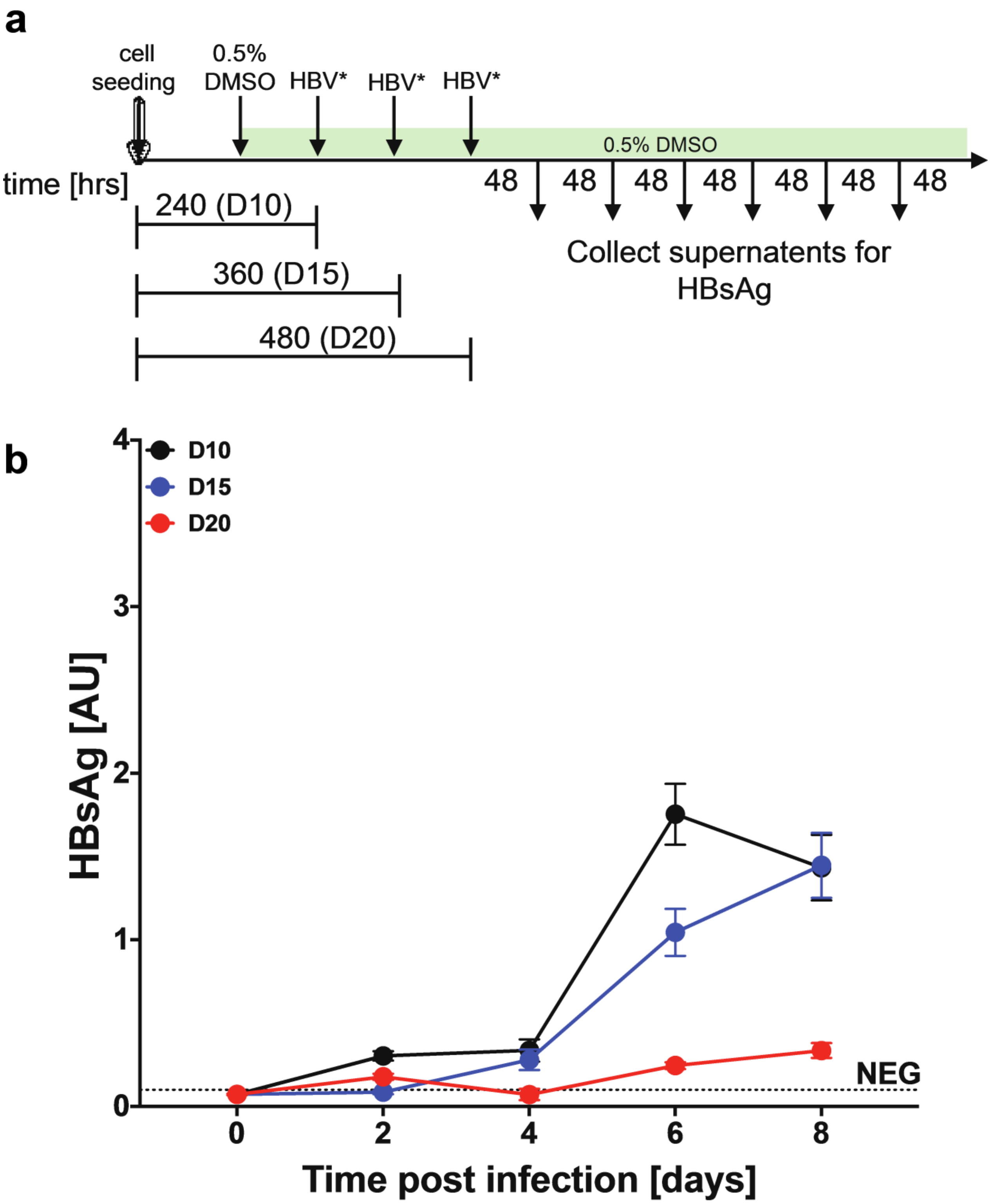

Supplementary Figure 4. SACC-PHHs remain robustly susceptible to HBV infection for up to 15 days post seeding. (**a**). Experimental time course. (**b**). HBsAg kinetic time course data for SACC-PHH (mixed donor HU1007) challenged at days 10 (black),15 (blue), or 20 (red) post seeding. For each time point four biological replicates were performed. All data are presented as means ± s.d.

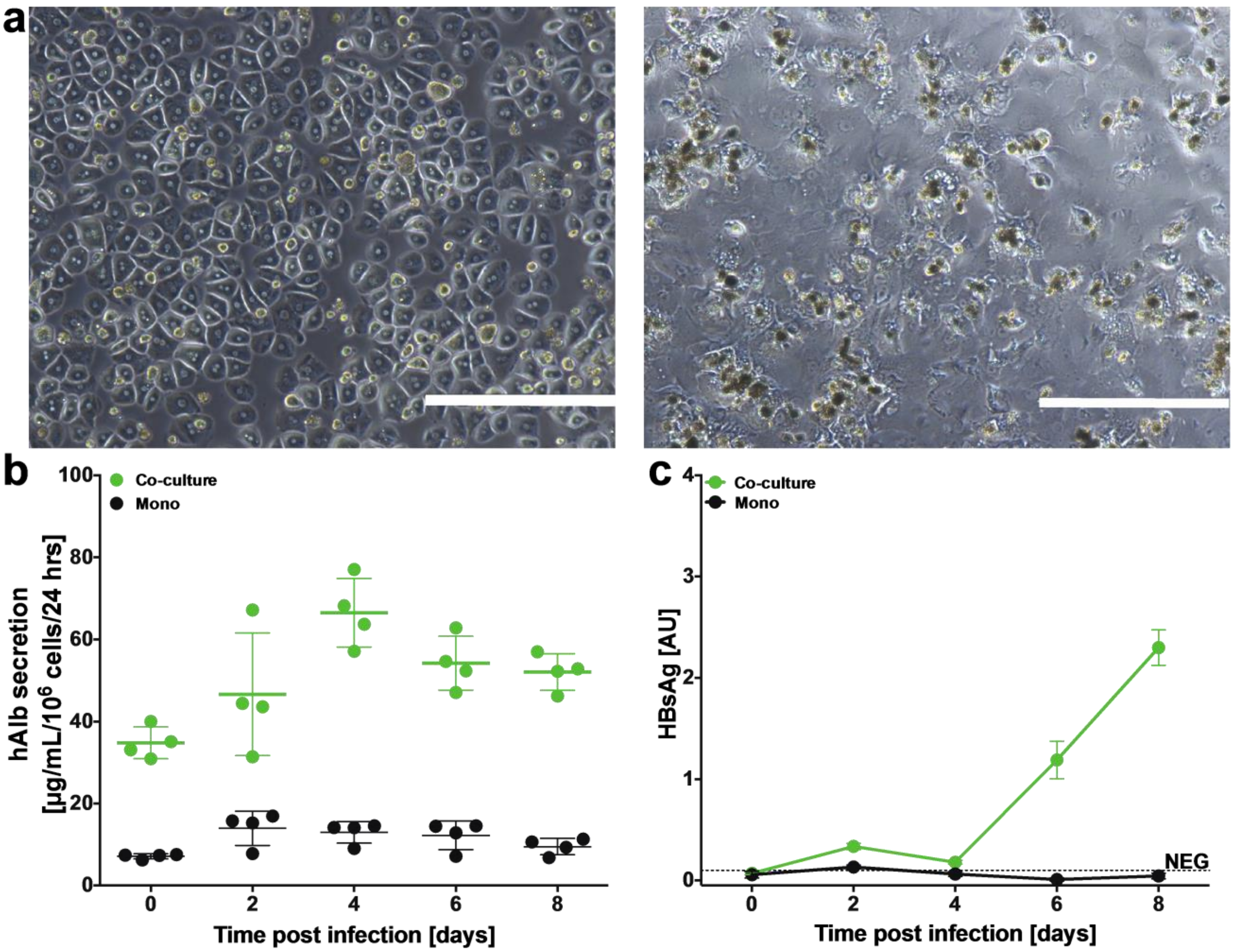

Supplementary Figure 5. SACC-PHH platform is responsible for prolonged hepatocyte health and susceptibility to HBV infection. (**a**). Bright field images of PHHs one day after plating (left) and 8 days (right), scale bar= 400 **µ**m. (**b**). hAlb concentration of HU1003 SACC-PHHs (green) and HU1003 monoculture (black). (**c**). HBsAg data for HU1003 SACC-PHHs (green) and HU1003 monoculture (black). For all time points four biological replicates were performed. All data are presented as means ± s.d.

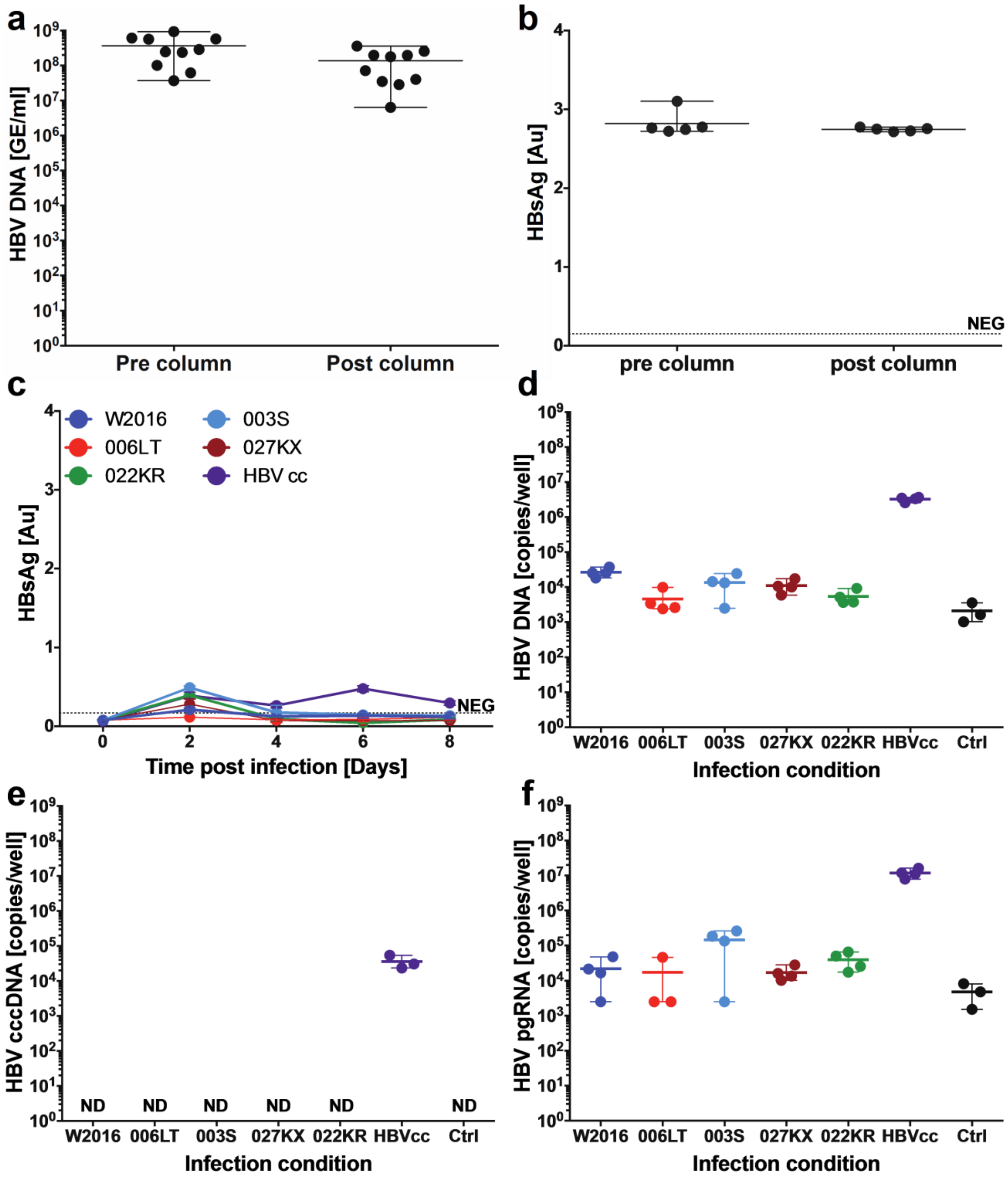

Supplementary Figure 6. Assessment of heparin column purification of HBV virus from patient plasma and challenge with said HBV of hNTCP-eGFP HepG2 cells. (**a**). HBV DNA quantification pre and post heparin column purification of HBVpat. (**b**). HBsAg quantification pre and post heparin column purification of HBVpat. (**c**) HBsAg quantification for hNTCP-eGFP HepG2 cells challenged with HBVcc and HBV from patient plasma. (**d**) Total HBV DNA quantification by qPCR for hNTCP-eGFP HepG2 cells challenged with heparin column purified HBVpat and HBVcc. (**e**) Quantification of HBV pgRNA for HBVcc and HBVpat challenged hNTCP-eGFP HepG2 cells. (**f**) HBV cccDNA quantification for hNTCP-eGFP HepG2 cells challenged with HBVcc or HBVpat. For heparin column purification five to ten biological replicates were performed. For HBVpat infections of 3B10 cells four biological replicates were performed. All data are presented as means ± s.d.

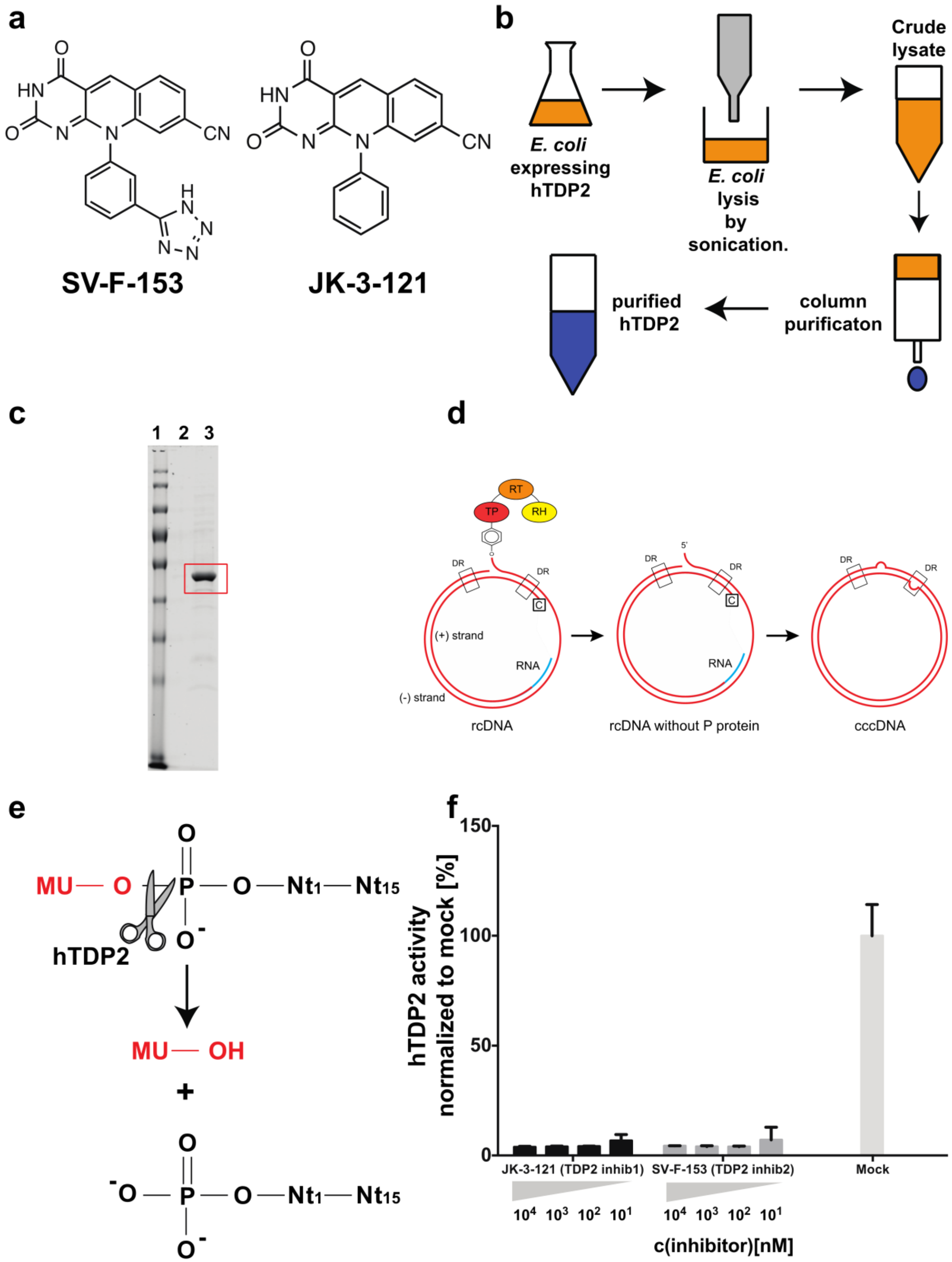

Supplementary Figure 7. hTDP2 is inhibited *in vitro* by small molecule hTDP2 inhibitors JK-3-121 and SV-F-153. (**a**). Structure of TDP2 inhibitor compounds JK-3-121 and SV-F-153. (**b**). Schematic of expression and purification procedure for hTDP2. (**c**). Coomassie gel of nickel column purified hTDP2. Lane 1= Page Rule MW ladder, Lane 2= Blank, Lane 3= pooled purified hTDP2 fractions. hTDP2 band boxed in red. (**d**). Schematic representation for theoretical role of hTDP2 in HBV viral polymerase removal. (**e**) Schematic representation of hTDP2 activity assay using MUP-DNA oligo and JK-3-121 and SV-F-153 hTDP2 inhibitors. (**f**) Normalized fluorescence data for *in vitro* inhibitory assay of hTDP2 activity with JK-3-121 and SV-F-153 inhibitors. For *in vitro* hTDP2 inhibition assays four replicates were performed. All data are presented as means ± s.d.

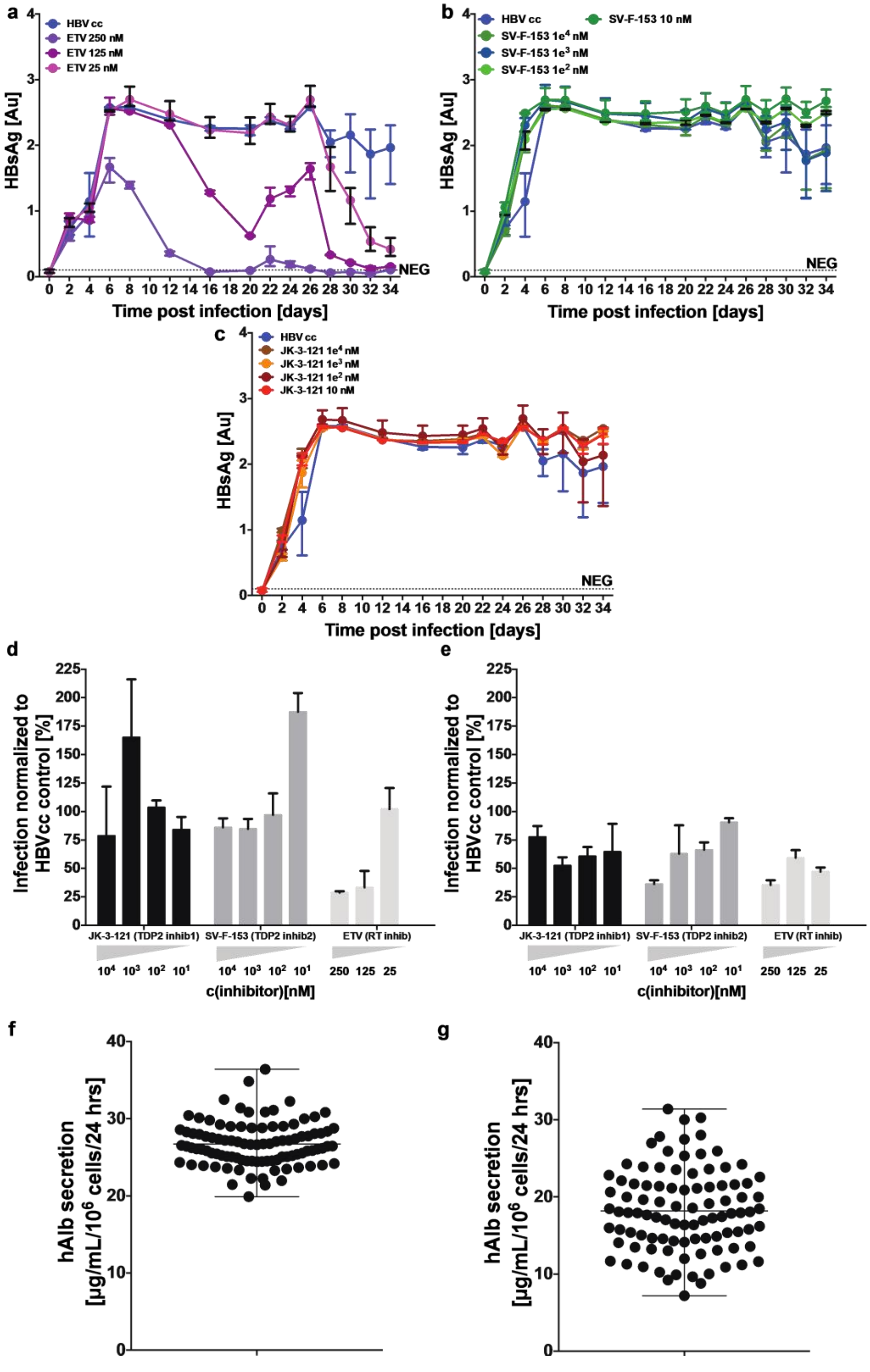

Supplementary Figure 8. Characterization of drug treatment in SACC-PHHs and 3B10 cells. (**a-c**). Assessment of prophylactic inhibition by ETV, JK-3-121, and SV-F-153 in SACC-PHH platform on HBsAg secretion over 34 days. (**a**). ETV, (**b**). SV-F-153, (**c**) JK-3-121. (**d**). Prophylactic treatment of 3B10 cells with JK-3-121, SV-F-153, and ETV (Day 14). X axis: concentration of different drugs. Y axis: Amount of HBsAg secretion normalized to that secreted by HBVcc infected untreated control cells. (**e**) Drug treatment of 3B10 cells with JK-3-121, SV-F-153, and ETV (Day 20). (**f**) hAlb quantification across 96 well plate of SACC-PHH's from prophylactic drug treatment (day 30; corresponds to **Figure 2j**). (**g**) hAlb quantification across a 96 well plate of SACC-PHHs from drug treatment experiment (day 30; corresponds to **Figure 2k**). For panels (**a-e**) four biological replicates were performed. For panels (**f-g**) 96 biological replicates. All data are presented as means ± s.d.

				Serological Testing						
**Human donor #**	**Age (years)**	**Gender**	**Race**	**EBV**	**RPR**	**CMV**	**Hep B**	**Hep C**	**HIV**	**Social History**
Mixed donor HU1007	Age range 23-69	Male (3), Female (2)	Caucasian (4), Arabic (1)	P(1), NR(4)	N(5)	P(2), N(3)	N(5)	N(5)	N(5)	Drug use (1), Heavy drinker (2), No Alcohol use (2)
Mixed donor HU1008	Age range 7-69	Male (4), Female (1)	Caucasian (3), Asian (2)	N/A	N/A	P(3), N(2)	N(5)	N(5)	N(5)	N/A
Single donor HU1002	54	Female	Hispanic	N/A	N	N	N	N	N	Drug use (N), Alcohol (N)
Single donor HU1003	64	Male	Caucasian	P	N	N	N	N	N	Drug use (N), Alcohol (N)
Single donor HU1004	38	Female	Caucasian	N/A	N/A	N	N	N	N	Drug use (N), Alcohol (N)
Single donor HU1010	52	N/A	Caucasian	P	N	P	N	N	N	Drug use (P), Alcohol (N)
Single donor HU1013	65	Male	Caucasian	P	N	P	N	N	N	N/A

Supplemental Table 1. Demographics, serological testing, and health history of mixed and single donor SACC-PHH's. Terms used: Epstein-Barr virus (EBV), Rapid plasma reagin (RPR), cytomegalovirus (CMV), hepatitis B virus (Hep B), hepatitis C virus (Hep C), human immunodeficiency virus (HIV), not applicable (N/A), not reported (NR), positive (P), and negative (N).

Enzyme (Substrate) [Concentration µM]	***Cytochrome P450 3A4 (Midazolam), [5]***	***Cytochrome P450 2D6 (Dextromethorphan), [20]***	***Cytochrome P450 2C9 (Tolbutamide), [20]***
**Mixed donor HU1008** (nmol/hr/10^6 cells)	Day 1 (2.263), Day 4(1.687), Day 8 (1.053)	Day 1 (0.759), Day 4(0.848), Day 8 (0.454)	Day 1 (0.556), Day 4(0.443), Day 8 (0.32)
**Mixed donor HU1007** (nmol/hr/10^6 cells)	Day 1 (2.487), Day 4(3.99), Day 8 (2.523)	Day 1 (2.137), Day 4(5.197), Day 8 (3.317)	Day 1 (0.396), Day 4(0.496), Day 8 (0.411)
**Single donor HU1010** (nmol/hr/10^6 cells)	Day 1 (1.03), Day 4(1.23), Day 8 (0.79)	Day 1 (0.528), Day 4(0.311), Day 8 (0.175)	Day 1 (0.137), Day 4(0.135), Day 8 (0.093)
**Single donor HU1004** (nmol/hr/10^6 cells)	Day 1 (0.29), Day 4(), Day 8 (0.216)	Day 1 (0.13), Day 4(), Day 8 (0.298)	Day 1 (0.223), Day 4(), Day 8 (0.266)
**Single donor HU1002** (nmol/hr/10^6 cells)	Day 1 (0.9895), Day 4(1.05), Day 8 (0.495)	Day 1 (0.452), Day 4(0.391), Day 8 (0.316)	Day 1 (0.059), Day 4(0.025), Day 8 (0.021)
**Single donor HU1003** (nmol/hr/10^6 cells)	Day 1 (0.425), Day 4(), Day 8 (0.777)	Day 1 (0.598), Day 4(), Day 8 (0.468)	Day 1 (0.0179), Day 4(), Day 8 (0.0131)
**Single donor HU1013** (nmol/hr/10^6 cells)	Day 1 (0.18), Day 4(0.248), Day 8 (0.131)	Day 1 (0.504), Day 4(0.248), Day 8 (0.21)	Day 1 (0.168), Day 4(0.1437), Day 8 (0.0757)

Supplementary Table 2. Characterization of enzymatic and metabolic function post plating for single and mixed donor SACC-PHHs.
